# Supplementary material for: Influenza A Virus Infection Reactivates Human Endogenous Retroviruses Associated with Modulation of Antiviral Immunity
Source: Viruses. 2022 Jul 21;14(7):1591. doi: 10.3390/v14071591 (PMC9320126; doi:10.3390/v14071591)
Supplement: Supplementary file 1 [file viruses-14-01591-s001.zip › MotifAnalysis.zip!/MotifOutput_PR8M/homerResults/motif4.similar.html]

motif4

## Information for motif4

A
C
T
G
A
C
G
T
A
G
T
C
C
G
T
A
A
C
T
G
A
C
T
G
A
G
T
C
A
G
T
C
A
G
T
C
A
C
G
T
A
G
T
C
A
G
T
C
  
Reverse Opposite:  

A
C
T
G
A
C
T
G
G
T
C
A
C
T
A
G
A
C
T
G
A
C
T
G
A
G
T
C
A
G
T
C
A
C
G
T
A
C
T
G
C
G
T
A
A
G
T
C
  

|  |  |
| --- | --- |
| p-value: | 1e-18 |
| log p-value: | -4.324e+01 |
| Information Content per bp: | 1.941 |
| Number of Target Sequences with motif | 5.0 |
| Percentage of Target Sequences with motif | 21.74% |
| Number of Background Sequences with motif | 1.3 |
| Percentage of Background Sequences with motif | 0.00% |
| Average Position of motif in Targets | 97.2 +/- 11.5bp |
| Average Position of motif in Background | 143.2 +/- 49.1bp |
| Strand Bias (log2 ratio + to - strand density) | -1.0 |
| Multiplicity (# of sites on avg that occur together) | 1.20 |
| Motif File: | file (matrix) reverse opposite |

### Similar de novo motifs found

|  |  |  |  |  |  |  |  |
| --- | --- | --- | --- | --- | --- | --- | --- |
| Rank | Match Score | Redundant Motif | P-value | log P-value | % of Targets | % of Background | Motif file |
| 1 | 0.663 | A C T G A C T G A G T C A G T C A C G T A C T G C G T A A G C T C G T A C G A T | 1e-14 | -33.808283 | 26.09% | 0.05% | motif file (matrix) |
| 2 | 0.773 | A G T C A T G C A C T G A G C T A G T C C G T A A C T G A C T G A G T C A G T C | 1e-9 | -22.932832 | 26.09% | 0.32% | motif file (matrix) |
| 3 | 0.610 | A G T C A T C G A C T G A T G C A G T C C G T A A C T G A C T G A G T C A G T C A C G T A C T G | 1e-9 | -21.421506 | 13.04% | 0.01% | motif file (matrix) |
| 4 | 0.842 | C T A G A C G T A G T C C G T A A C T G A C T G A G T C A G T C | 1e-6 | -15.101281 | 21.74% | 0.62% | motif file (matrix) |
